# 2-(2-Chloro-6,7-dimethyl­quinolin-3-yl)-2,3-dihydro­quinolin-4(1*H*)-one

**DOI:** 10.1107/S1600536811028170

**Published:** 2011-07-23

**Authors:** Saida Benzerka, Abdelmalek Bouraiou, Sofiane Bouacida, Thierry Roisnel, Ali Belfaitah

**Affiliations:** a Laboratoire des Produits Naturels d’Origine Végétale et de Synthèse Organique, PHYSYNOR, Université Mentouri-Constantine, 25000 Constantine, Algeria; b Unité de Recherche de Chimie de l’Environnement et Moléculaire Structurale, CHEMS, Université Mentouri-Constantine, 25000 Algeria; c Centre de difractométrie X, UMR 6226 CNRS Unité Sciences Chimiques de Rennes, Université de Rennes I, 263 Avenue du Général Leclerc, 35042 Rennes, France

## Abstract

In the title mol­ecule, C_20_H_17_ClN_2_O, the dihedral angle between the mean plane of the quinoline ring system and the benzene ring of the dihydro­quinolinone moiety is 57.84 (8)°. In the crystal, mol­ecules are linked into centrosymmetric dimers *via* pairs of inter­molecular N—H⋯N hydrogen bonds. These dimers are further stabilized by weak π–π stacking inter­actions between pyridine rings with a centroid–centroid distance of 3.9414 (12) Å.

## Related literature

For quinoline compounds and their applications, see: Prakash *et al.* (1994 ▶); Singh & Kapil (1993 ▶); Kalinin *et al.* (1992 ▶); Xia *et al.* (1992 ▶); Donnelly & Farrell (1990*a* ▶,*b* ▶); Kumar *et al.* (2004 ▶); Varma & Saini (1997 ▶); Tokes & Litkei (1993 ▶); Tokes & Szilagyi (1987 ▶); Tokes *et al.* (1992 ▶). For our previous work on quinoline derivatives, see: Belfaitah *et al.* (2006 ▶); Bouraiou *et al.* (2008 ▶, 2010 ▶, 2011 ▶); Benzerka *et al.* (2010 ▶); Ladraa *et al.* (2010 ▶). 
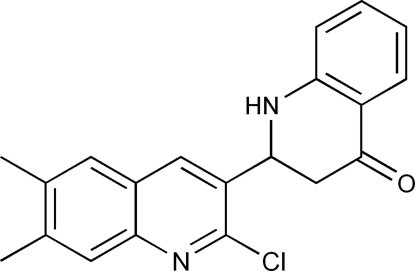


## Experimental

### 

#### Crystal data

C_20_H_17_ClN_2_O
*M* *_r_* = 336.81
Triclinic, 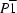

*a* = 7.7345 (4) Å
*b* = 10.6196 (6) Å
*c* = 11.3463 (4) Å
α = 96.425 (2)°
β = 100.068 (3)°
γ = 109.576 (1)°
*V* = 849.84 (7) Å^3^
*Z* = 2
Mo *K*α radiation
μ = 0.23 mm^−1^
*T* = 295 K
0.15 × 0.06 × 0.05 mm

#### Data collection

Nonius KappaCCD diffractometer
7058 measured reflections
3863 independent reflections
2507 reflections with *I* > 2σ(*I*)
*R*_int_ = 0.029

#### Refinement

*R*[*F* ^2^ > 2σ(*F* ^2^)] = 0.048
*wR*(*F* ^2^) = 0.133
*S* = 1.00
3863 reflections
222 parameters
H atoms treated by a mixture of independent and constrained refinement
Δρ_max_ = 0.17 e Å^−3^
Δρ_min_ = −0.20 e Å^−3^

### 

Data collection: *COLLECT* (Nonius, 1998 ▶); cell refinement: *SCALEPACK* (Otwinowski & Minor, 1997 ▶); data reduction: *DENZO* (Otwinowski & Minor, 1997 ▶) and *SCALEPACK*; program(s) used to solve structure: *SIR2002* (Burla *et al.*, 2005 ▶); program(s) used to refine structure: *SHELXL97* (Sheldrick, 2008 ▶); molecular graphics: *ORTEP-3* (Farrugia, 1997 ▶) and *DIAMOND* (Brandenburg & Berndt, 2001 ▶); software used to prepare material for publication: *WinGX* (Farrugia, 1999 ▶).

## Supplementary Material

Crystal structure: contains datablock(s) global. DOI: 10.1107/S1600536811028170/lh5284sup1.cif

Supplementary material file. DOI: 10.1107/S1600536811028170/lh5284globalsup2.cml

Additional supplementary materials:  crystallographic information; 3D view; checkCIF report

## Figures and Tables

**Table 1:** Hydrogen-bond geometry (Å, °)

*D*—H⋯*A*	*D*—H	H⋯*A*	*D*⋯*A*	*D*—H⋯*A*
N2—H2*N*⋯N1^i^	0.86 (2)	2.53 (2)	3.297 (2)	148.6 (18)

Symmetry code: (i) 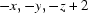.

**Fig. 1.**
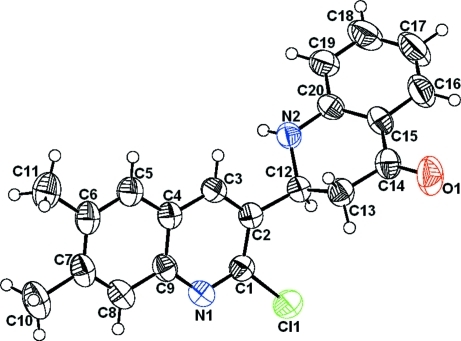
The molecular structure of the title compound with displacement ellipsoids drawn at the 50% probability level.

**Fig. 2.**
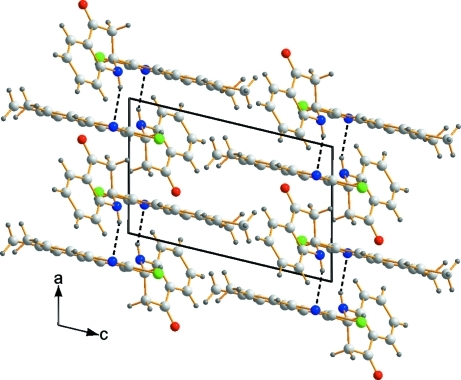
Part of the crytal structure viewed along the b axis showing hydrogen bonds as dashed lines.

## supplementary crystallographic information

### Comment

2-Phenyl-2,3-dihydroquinolin-4(1*H*)-one compound substituted on the aromatic rings are valuable precursors (Prakash *et al.*, 1994; Singh & Kapil, 1993) for the synthesis of medicinally important compounds, which are often not readily accessible by other means (Kalinin *et al.*, 1992; Xia *et al.*, 1992). The formation of 2,3-dihydroquinolin-4(1*H*)-ones is generally accomplished by acid- or base-catalyzed isomerization of substituted 2'-aminochalcones (Donnelly & Farrell, 1990*a*,*b*; Tokes & Litkei, 1993). Most of the procedures involve the use of corrosive reagents such as orthophosphoric acid, acetic acid or strong alkali. Many attempts have been made to explore efficient catalysts to accelerate this kind of reaction. Some of them are of limited synthetic scope due to low yields, long reaction times and the need for large amount of catalyst, specialized solvents or microwave activation (Tokes & Szilagyi, 1987; Tokes *et al.*, 1992; Kumar *et al.* 2004; Varma & Saini, 1997). In continuation of our studies on quinoline derivatives and their biological activities (Bouraiou *et al.*, 2010; Benzerka *et al.*, 2010; Ladraa *et al.*, 2010) we report herein the synthesis and structure determination of 2-(2-chloro-6,7-dimethylquinolin-3-yl)-2,3-dihydroquinolin-4(1*H*)-one I (Bouraiou *et al.*, 2011). Characterization of the compound I was made from its spectral data (^1^H-NMR, ^13^C-NMR), and was unequivocally established from an X-ray crystallographic determination (I).

The molecular structure of (I) is shown in Fig. 1. The two rings of quinolyl moiety are fused in an axial fashion and form a dihedral angle of 0.28 (7)° and this quasi plane system forms dihedral angles of 57.84 (8)° with the benzene ring (C15-C20). The geometric parameters of (I) are in agreement with those of other structures possessing a quinolyl substituent previously reported in the literature (Belfaitah *et al.*, 2006; Bouraiou *et al.*, 2008; Bouraiou *et al.*, 2011). In the crystal, molecules are linked into centrosymmetric dimers via pairs of intermolecular N–H···N hydrogen bonds (Fig. 2). These dimers are further stabilized by π–π stacking interactions between pyridine rings with a centroid to centroid distance of 3.9414 (12)Å.

### Experimental

A mixture of (E)-1-(2-aminophenyl)-3-(2-chloro-6,7-dimethylquinolin-3-yl)prop-2-en-1-one and silica gel (1 g) impregnated with indium (III) chloride (20 mol%) was irradiated in domestic microwave oven at 360 W for 5 minutes (Bouraiou *et al.*, 2011). Under these conditions, compound (I) was successfully synthesized in good yield (63%). A suitable crystal of title compound were obtained by crystallization from a CH_2_Cl_2_/di-isopropylether solution.

### Refinement

All H atoms bonded to C atoms were located in difference Fourier maps but were introduced in calculated positions and treated as riding with C—H = 0.93-0.97Å and U_iso_(H) = 1.2U_eq_(C) or 1.5U_eq_(C) for methyl groups. The H atom boned to N2 was refined independently with U_iso_(H) = 1.2U_eq_(N).

### Crystal data

**Table:**

C_20_H_17_ClN_2_O	*Z* = 2
*M**_r_* = 336.81	*F*(000) = 352
Triclinic, *P*1	*D*_x_ = 1.316 Mg m^−^^3^
*a* = 7.7345 (4) Å	Mo *K*α radiation, λ = 0.71073 Å
*b* = 10.6196 (6) Å	Cell parameters from 3734 reflections
*c* = 11.3463 (4) Å	θ = 2.9–27.5°
α = 96.425 (2)°	µ = 0.23 mm^−^^1^
β = 100.068 (3)°	*T* = 295 K
γ = 109.576 (1)°	Needle, white
*V* = 849.84 (7) Å^3^	0.15 × 0.06 × 0.05 mm

### Data collection

**Table:**

Nonius KappaCCD diffractometer	2507 reflections with *I* > 2σ(*I*)
Radiation source: Enraf–Nonius FR590	*R*_int_ = 0.029
graphite	θ_max_ = 27.5°, θ_min_ = 3.0°
Detector resolution: 9 pixels mm^-1^	*h* = −10→10
CCD rotation images, thick slices scans	*k* = −13→13
7058 measured reflections	*l* = −14→14
3863 independent reflections	

### Refinement

**Table:**

Refinement on *F*^2^	Primary atom site location: structure-invariant direct methods
Least-squares matrix: full	Secondary atom site location: difference Fourier map
*R*[*F*^2^ > 2σ(*F*^2^)] = 0.048	Hydrogen site location: inferred from neighbouring sites
*wR*(*F*^2^) = 0.133	H atoms treated by a mixture of independent and constrained refinement
*S* = 1.00	*w* = 1/[σ^2^(*F*_o_^2^) + (0.0621*P*)^2^ + 0.0886*P*] where *P* = (*F*_o_^2^ + 2*F*_c_^2^)/3
3863 reflections	(Δ/σ)_max_ < 0.001
222 parameters	Δρ_max_ = 0.17 e Å^−^^3^
0 restraints	Δρ_min_ = −0.20 e Å^−^^3^

### Special details

**Table:**

Geometry. All e.s.d.'s (except the e.s.d. in the dihedral angle between two l.s. planes) are estimated using the full covariance matrix. The cell e.s.d.'s are taken into account individually in the estimation of e.s.d.'s in distances, angles and torsion angles; correlations between e.s.d.'s in cell parameters are only used when they are defined by crystal symmetry. An approximate (isotropic) treatment of cell e.s.d.'s is used for estimating e.s.d.'s involving l.s. planes.
Refinement. Refinement of *F*^2^ against ALL reflections. The weighted *R*-factor *wR* and goodness of fit *S* are based on *F*^2^, conventional *R*-factors *R* are based on *F*, with *F* set to zero for negative *F*^2^. The threshold expression of *F*^2^ > σ(*F*^2^) is used only for calculating *R*-factors(gt) *etc*. and is not relevant to the choice of reflections for refinement. *R*-factors based on *F*^2^ are statistically about twice as large as those based on *F*, and *R*- factors based on ALL data will be even larger.

### Fractional atomic coordinates and isotropic or equivalent isotropic displacement parameters (Å^2^)

**Table:**

	*x*	*y*	*z*	*U*_iso_*/*U*_eq_	
C1	0.2476 (3)	−0.00341 (19)	0.99805 (16)	0.0463 (4)	
C2	0.2561 (2)	0.13287 (18)	1.01932 (15)	0.0442 (4)	
C3	0.2601 (3)	0.18540 (19)	1.13579 (16)	0.0474 (4)	
H3	0.2659	0.2744	1.1546	0.057*	
C4	0.2556 (3)	0.10607 (19)	1.22794 (16)	0.0475 (4)	
C5	0.2595 (3)	0.1535 (2)	1.35012 (17)	0.0549 (5)	
H5	0.2666	0.2423	1.3728	0.066*	
C6	0.2531 (3)	0.0720 (2)	1.43636 (17)	0.0578 (5)	
C7	0.2407 (3)	−0.0639 (2)	1.40197 (18)	0.0579 (5)	
C8	0.2379 (3)	−0.1118 (2)	1.28402 (18)	0.0558 (5)	
H8	0.2314	−0.2007	1.2624	0.067*	
C9	0.2446 (2)	−0.02881 (19)	1.19481 (16)	0.0476 (4)	
C10	0.2307 (4)	−0.1569 (3)	1.4943 (2)	0.0806 (7)	
H10A	0.2206	−0.2449	1.4553	0.121*	
H10B	0.1225	−0.1653	1.5278	0.121*	
H10C	0.3427	−0.1194	1.5584	0.121*	
C11	0.2620 (4)	0.1278 (3)	1.56687 (18)	0.0767 (7)	
H11A	0.3749	0.1284	1.6184	0.115*	
H11B	0.1542	0.0716	1.5921	0.115*	
H11C	0.2625	0.2188	1.5729	0.115*	
C12	0.2648 (2)	0.21733 (18)	0.91943 (16)	0.0448 (4)	
H12	0.1964	0.1569	0.8412	0.054*	
C13	0.4673 (3)	0.2922 (2)	0.91241 (18)	0.0535 (5)	
H13A	0.5382	0.3471	0.9914	0.064*	
H13B	0.5245	0.2265	0.8924	0.064*	
C14	0.4785 (3)	0.3822 (2)	0.81848 (18)	0.0566 (5)	
C15	0.3261 (3)	0.43479 (19)	0.79376 (17)	0.0524 (5)	
C16	0.3250 (4)	0.5217 (2)	0.7094 (2)	0.0738 (7)	
H16	0.4184	0.5419	0.6653	0.089*	
C17	0.1893 (4)	0.5769 (3)	0.6911 (3)	0.0911 (9)	
H17	0.1905	0.6342	0.6348	0.109*	
C18	0.0495 (4)	0.5478 (3)	0.7563 (3)	0.0833 (8)	
H18	−0.0425	0.5862	0.7438	0.1*	
C19	0.0459 (3)	0.4632 (2)	0.8389 (2)	0.0609 (5)	
H19	−0.0483	0.4446	0.8824	0.073*	
C20	0.1829 (3)	0.40416 (18)	0.85847 (16)	0.0480 (4)	
N1	0.2392 (2)	−0.08277 (15)	1.07803 (14)	0.0498 (4)	
N2	0.1772 (2)	0.31720 (17)	0.94168 (14)	0.0488 (4)	
H2N	0.066 (3)	0.284 (2)	0.9549 (18)	0.059*	
O1	0.6107 (2)	0.4116 (2)	0.76829 (17)	0.0858 (5)	
Cl1	0.24838 (8)	−0.07686 (5)	0.85241 (5)	0.06466 (19)	

### Atomic displacement parameters (Å^2^)

**Table:**

	*U*^11^	*U*^22^	*U*^33^	*U*^12^	*U*^13^	*U*^23^
C1	0.0434 (10)	0.0469 (10)	0.0467 (9)	0.0148 (8)	0.0099 (7)	0.0071 (7)
C2	0.0408 (9)	0.0471 (10)	0.0440 (9)	0.0149 (8)	0.0086 (7)	0.0108 (7)
C3	0.0513 (11)	0.0436 (10)	0.0484 (9)	0.0177 (8)	0.0121 (8)	0.0101 (8)
C4	0.0465 (10)	0.0507 (11)	0.0448 (9)	0.0160 (8)	0.0106 (8)	0.0115 (8)
C5	0.0545 (12)	0.0603 (12)	0.0492 (10)	0.0194 (10)	0.0121 (9)	0.0114 (9)
C6	0.0499 (11)	0.0743 (14)	0.0460 (10)	0.0165 (10)	0.0108 (8)	0.0172 (9)
C7	0.0469 (11)	0.0706 (14)	0.0560 (11)	0.0163 (10)	0.0106 (9)	0.0281 (10)
C8	0.0523 (11)	0.0522 (12)	0.0602 (12)	0.0141 (9)	0.0088 (9)	0.0210 (9)
C9	0.0416 (10)	0.0491 (11)	0.0497 (10)	0.0124 (8)	0.0087 (8)	0.0150 (8)
C10	0.0827 (17)	0.0918 (19)	0.0721 (14)	0.0277 (14)	0.0192 (12)	0.0446 (13)
C11	0.0823 (17)	0.0991 (19)	0.0475 (11)	0.0304 (15)	0.0157 (11)	0.0156 (12)
C12	0.0453 (10)	0.0455 (10)	0.0436 (9)	0.0153 (8)	0.0107 (7)	0.0113 (7)
C13	0.0458 (10)	0.0588 (12)	0.0592 (11)	0.0198 (9)	0.0144 (9)	0.0170 (9)
C14	0.0492 (11)	0.0569 (12)	0.0599 (11)	0.0110 (9)	0.0169 (9)	0.0144 (9)
C15	0.0515 (11)	0.0424 (10)	0.0562 (11)	0.0070 (9)	0.0107 (9)	0.0144 (8)
C16	0.0760 (16)	0.0627 (14)	0.0876 (16)	0.0178 (12)	0.0308 (13)	0.0370 (12)
C17	0.102 (2)	0.0756 (18)	0.114 (2)	0.0372 (16)	0.0319 (17)	0.0595 (16)
C18	0.0805 (17)	0.0698 (16)	0.115 (2)	0.0385 (14)	0.0229 (15)	0.0436 (15)
C19	0.0586 (12)	0.0517 (12)	0.0761 (13)	0.0229 (10)	0.0156 (10)	0.0171 (10)
C20	0.0470 (10)	0.0381 (9)	0.0519 (10)	0.0095 (8)	0.0060 (8)	0.0073 (8)
N1	0.0514 (9)	0.0441 (9)	0.0530 (8)	0.0159 (7)	0.0107 (7)	0.0121 (7)
N2	0.0466 (9)	0.0510 (9)	0.0541 (9)	0.0193 (7)	0.0164 (7)	0.0177 (7)
O1	0.0641 (10)	0.1091 (14)	0.0992 (12)	0.0288 (10)	0.0431 (9)	0.0487 (11)
Cl1	0.0817 (4)	0.0589 (3)	0.0535 (3)	0.0264 (3)	0.0186 (2)	0.0028 (2)

### Geometric parameters (Å, °)

**Table:**

C1—N1	1.301 (2)	C11—H11B	0.96
C1—C2	1.418 (3)	C11—H11C	0.96
C1—Cl1	1.7485 (19)	C12—N2	1.459 (2)
C2—C3	1.367 (2)	C12—C13	1.521 (3)
C2—C12	1.519 (2)	C12—H12	0.98
C3—C4	1.412 (2)	C13—C14	1.504 (3)
C3—H3	0.93	C13—H13A	0.97
C4—C9	1.410 (3)	C13—H13B	0.97
C4—C5	1.413 (3)	C14—O1	1.223 (2)
C5—C6	1.373 (3)	C14—C15	1.463 (3)
C5—H5	0.93	C15—C20	1.403 (3)
C6—C7	1.420 (3)	C15—C16	1.403 (3)
C6—C11	1.513 (3)	C16—C17	1.360 (4)
C7—C8	1.371 (3)	C16—H16	0.93
C7—C10	1.512 (3)	C17—C18	1.385 (4)
C8—C9	1.411 (2)	C17—H17	0.93
C8—H8	0.93	C18—C19	1.367 (3)
C9—N1	1.371 (2)	C18—H18	0.93
C10—H10A	0.96	C19—C20	1.400 (3)
C10—H10B	0.96	C19—H19	0.93
C10—H10C	0.96	C20—N2	1.389 (2)
C11—H11A	0.96	N2—H2N	0.86 (2)
			
N1—C1—C2	126.09 (17)	H11B—C11—H11C	109.5
N1—C1—Cl1	114.81 (14)	N2—C12—C2	110.34 (14)
C2—C1—Cl1	119.11 (13)	N2—C12—C13	108.54 (15)
C3—C2—C1	116.21 (16)	C2—C12—C13	111.35 (15)
C3—C2—C12	121.63 (17)	N2—C12—H12	108.9
C1—C2—C12	122.15 (16)	C2—C12—H12	108.9
C2—C3—C4	120.78 (17)	C13—C12—H12	108.9
C2—C3—H3	119.6	C14—C13—C12	111.87 (16)
C4—C3—H3	119.6	C14—C13—H13A	109.2
C9—C4—C3	117.57 (16)	C12—C13—H13A	109.2
C9—C4—C5	118.52 (16)	C14—C13—H13B	109.2
C3—C4—C5	123.91 (18)	C12—C13—H13B	109.2
C6—C5—C4	121.8 (2)	H13A—C13—H13B	107.9
C6—C5—H5	119.1	O1—C14—C15	122.46 (19)
C4—C5—H5	119.1	O1—C14—C13	121.3 (2)
C5—C6—C7	119.29 (18)	C15—C14—C13	116.24 (17)
C5—C6—C11	120.1 (2)	C20—C15—C16	118.8 (2)
C7—C6—C11	120.59 (19)	C20—C15—C14	120.37 (17)
C8—C7—C6	119.87 (17)	C16—C15—C14	120.79 (19)
C8—C7—C10	119.5 (2)	C17—C16—C15	121.0 (2)
C6—C7—C10	120.6 (2)	C17—C16—H16	119.5
C7—C8—C9	121.2 (2)	C15—C16—H16	119.5
C7—C8—H8	119.4	C16—C17—C18	120.1 (2)
C9—C8—H8	119.4	C16—C17—H17	119.9
N1—C9—C4	122.10 (15)	C18—C17—H17	119.9
N1—C9—C8	118.61 (18)	C19—C18—C17	120.4 (2)
C4—C9—C8	119.29 (17)	C19—C18—H18	119.8
C7—C10—H10A	109.5	C17—C18—H18	119.8
C7—C10—H10B	109.5	C18—C19—C20	120.5 (2)
H10A—C10—H10B	109.5	C18—C19—H19	119.7
C7—C10—H10C	109.5	C20—C19—H19	119.7
H10A—C10—H10C	109.5	N2—C20—C19	120.17 (18)
H10B—C10—H10C	109.5	N2—C20—C15	120.66 (18)
C6—C11—H11A	109.5	C19—C20—C15	119.16 (18)
C6—C11—H11B	109.5	C1—N1—C9	117.23 (16)
H11A—C11—H11B	109.5	C20—N2—C12	115.66 (15)
C6—C11—H11C	109.5	C20—N2—H2N	111.0 (14)
H11A—C11—H11C	109.5	C12—N2—H2N	114.5 (15)
			
N1—C1—C2—C3	1.6 (3)	N2—C12—C13—C14	−55.2 (2)
Cl1—C1—C2—C3	−178.31 (14)	C2—C12—C13—C14	−176.88 (16)
N1—C1—C2—C12	−179.75 (18)	C12—C13—C14—O1	−153.6 (2)
Cl1—C1—C2—C12	0.3 (2)	C12—C13—C14—C15	28.1 (2)
C1—C2—C3—C4	−0.1 (3)	O1—C14—C15—C20	−176.5 (2)
C12—C2—C3—C4	−178.73 (16)	C13—C14—C15—C20	1.8 (3)
C2—C3—C4—C9	−0.7 (3)	O1—C14—C15—C16	0.5 (3)
C2—C3—C4—C5	179.93 (18)	C13—C14—C15—C16	178.8 (2)
C9—C4—C5—C6	0.1 (3)	C20—C15—C16—C17	0.7 (4)
C3—C4—C5—C6	179.40 (19)	C14—C15—C16—C17	−176.3 (2)
C4—C5—C6—C7	−0.5 (3)	C15—C16—C17—C18	0.1 (4)
C4—C5—C6—C11	178.64 (19)	C16—C17—C18—C19	−0.3 (5)
C5—C6—C7—C8	0.9 (3)	C17—C18—C19—C20	−0.3 (4)
C11—C6—C7—C8	−178.26 (19)	C18—C19—C20—N2	−179.4 (2)
C5—C6—C7—C10	−179.1 (2)	C18—C19—C20—C15	1.1 (3)
C11—C6—C7—C10	1.7 (3)	C16—C15—C20—N2	179.17 (19)
C6—C7—C8—C9	−0.8 (3)	C14—C15—C20—N2	−3.8 (3)
C10—C7—C8—C9	179.2 (2)	C16—C15—C20—C19	−1.3 (3)
C3—C4—C9—N1	0.3 (3)	C14—C15—C20—C19	175.74 (18)
C5—C4—C9—N1	179.64 (17)	C2—C1—N1—C9	−2.1 (3)
C3—C4—C9—C8	−179.37 (17)	Cl1—C1—N1—C9	177.84 (13)
C5—C4—C9—C8	0.0 (3)	C4—C9—N1—C1	1.1 (3)
C7—C8—C9—N1	−179.27 (18)	C8—C9—N1—C1	−179.28 (17)
C7—C8—C9—C4	0.4 (3)	C19—C20—N2—C12	153.97 (18)
C3—C2—C12—N2	−30.9 (2)	C15—C20—N2—C12	−26.5 (2)
C1—C2—C12—N2	150.56 (17)	C2—C12—N2—C20	177.70 (15)
C3—C2—C12—C13	89.7 (2)	C13—C12—N2—C20	55.4 (2)
C1—C2—C12—C13	−88.8 (2)		

### Hydrogen-bond geometry (Å, °)

**Table:**

*D*—H···*A*	*D*—H	H···*A*	*D*···*A*	*D*—H···*A*
N2—H2N···N1^i^	0.86 (2)	2.53 (2)	3.297 (2)	148.6 (18)

Symmetry codes: (i) −*x*, −*y*, −*z*+2.
